# Plastic zone range of a roadway considering the creep effect

**DOI:** 10.1038/s41598-020-77384-5

**Published:** 2020-11-23

**Authors:** Haidong Chen, Xiangjun Chen, Zhaofeng Wang, Zhiqiang Li, Fenghua An

**Affiliations:** 1 School of Safety Science and Engineering, Henan Polytechnic University, Jiaozuo, 454000 Henan China; 2 Henan Key Laboratory of Coal Green Conversion, Henan Polytechnic University, Jiaozuo, 454000 Henan China; 3 Collaborative Innovation Center of Coal Safety Production of Henan Province, Jiaozuo, 454000 Henan China

**Keywords:** Solid Earth sciences, Energy science and technology

## Abstract

The plastic zone range is an important parameter in the analysis of damage characteristics and the degree of damage to the rock surrounding a roadway. Based on the establishment of a plastic zone calculation model considering the creep effect, this paper obtains the characteristics of the change in the plastic zone damage range with time by solving the model. Additionally, the validity of the model is verified by field experiments. The research results can provide guidance for gas pressure measurement and gas drainage in coal mines.

## Introduction

Gas disasters constitute the primary threat to safe production in many coal mines^1,2^. On the one hand, determining the damage range of the plastic zone of the roadway can improve the accuracy of measuring the gas pressure (an important index in predicting the risk of coal and gas outbursts) in a coal mine; on the other hand, it can improve the sealing effect of the gas drainage borehole, thereby improving the gas drainage rate of the coal seam^3–6^. Therefore, clearly establishing the damage range of the plastic zone of the roadway can indirectly prevent the occurrence of coal mine gas disasters.

Many scholars have carried out research on the plastic zone of a roadway. Galin^7^ considered that the shape of the plastic zone around the roadway in the uniform stress field is circular, and the formula for calculating the circular plastic zone was derived. Additionally, Chen et al.^8^ established the boundary implicit equation for the plastic zone of a circular roadway. Abdel et al.^9^, Leitman and Villaggio^10^ considered that the shape of the plastic zone of roadways located in non-uniform stress fields should be elliptical. Guo et al.^11^ and Wang et al.^12^ pointed out that the shape of the plastic zone of a roadway could be circular, elliptical or butterfly-shaped under different in situ stress conditions. Zhou and Wu^13^, Yang et al.^14^ used a numerical simulation method to study the plastic zone range of a roadway under different stress conditions.

The practice of mining shows that at constant stress, the creep of coal and rock will occur as time goes on; that is, the deformation of coal and rock will continue to grow with time^15–19^. Even under low stress conditions, the creep of coal and rock would also occur. Due to the creep effect, the plastic zone of a roadway will increase with time and finally approach a certain value. It can be seen that the previous research on the plastic zone of a roadway did not consider the creep effect of the surrounding rock of the roadway and thus cannot better guide engineering practice. In this paper, a plastic zone calculation model considering the creep effect was established to analyze the variation in the plastic zone damage range with time. The validity of the model is also verified by field experiments.

## Calculation model of the plastic zone of a roadway considering the creep effect

### Model assumptions

**Figure 1:**
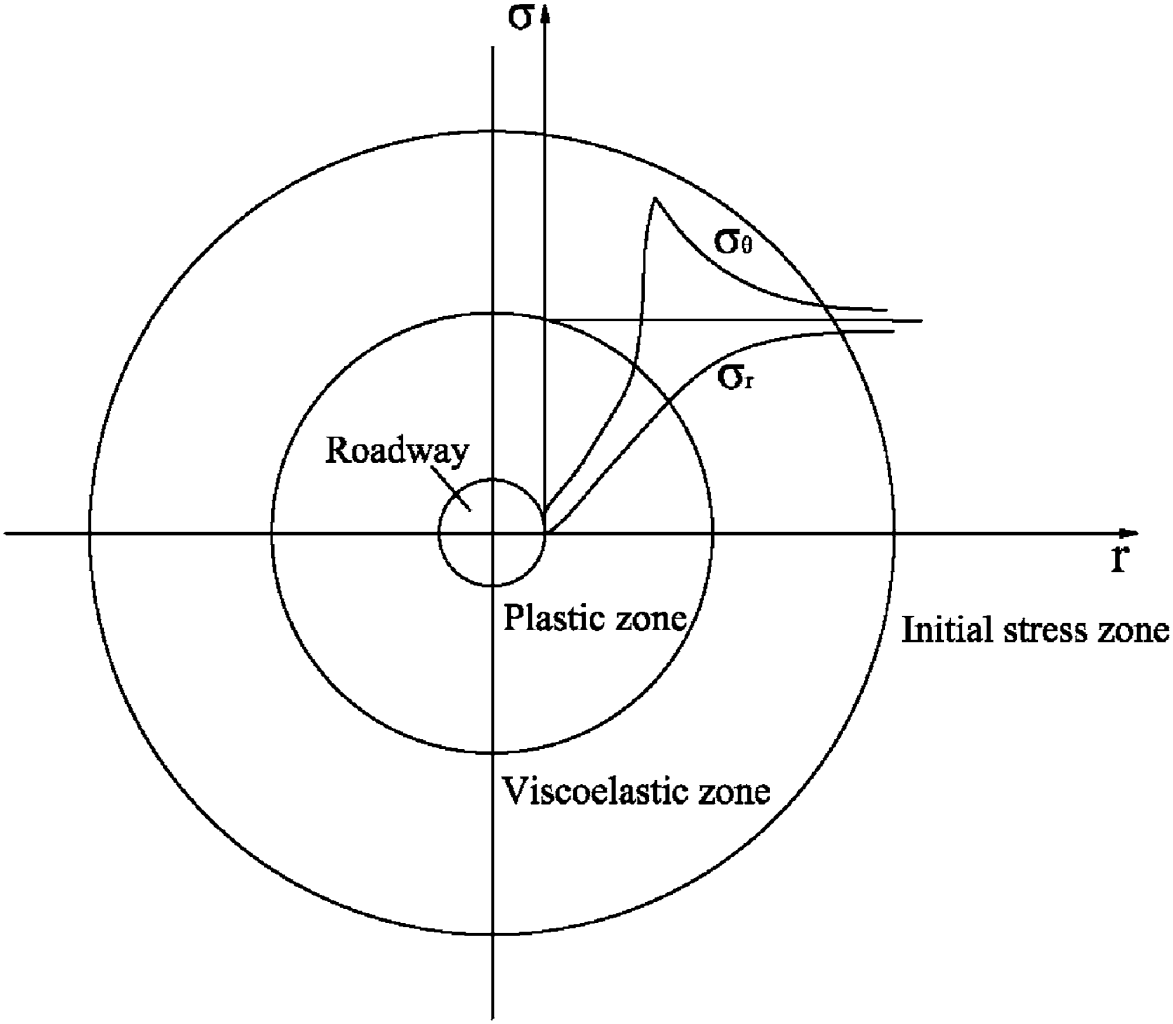
Stress distribution around the roadway^10,20^.

To obtain the specific range of the plastic zone of a roadway, some necessary practical assumptions are made to simplify the solution. The basic assumptions are as follows:

1. Since the axial length of the roadway is much larger than the radial width, the stress problem of the roadway section can be regarded as a plane strain problem.
2. The stress around the roadway is assumed to be uniform and the shape of the plastic zone of the roadway is assumed to be circular.
3. The failure of the plastic zone of the rock mass meets the Mohr–Coulomb yield criterion.
4. The stress distribution of any roadway section is shown in Fig. 1. Figure 1 shows the plastic zone, viscoelastic zone and initial stress zone.

### Calculation model of the plastic zone of a roadway

1. Stress distribution in the plastic zone

When the roadway is in the plane strain state, the static equilibrium equation of the plastic zone can be expressed as follows.

1 $$ \frac{{d\sigma_{r}^{p} }}{dr} + \frac{{\sigma_{r}^{p} - \sigma_{\theta }^{p} }}{r} = 0 $$

where $\sigma_{r}^{p}$ is the radial stress of the plastic zone of the roadway; $\sigma_{\theta }^{p}$ is the tangential stress of the plastic zone of the roadway; and $r$ is the radius of the surrounding rock from the center of the roadway.

According to Mohr–Coulomb plastic conditions, Eq. (2) can be obtained.

2 $$ \sigma_{\theta }^{p} = \frac{1 + \sin \varphi }{{1 - \sin \varphi }}\sigma_{r}^{p} + \frac{2C\cos \varphi }{{1 - \sin \varphi }} $$

where $\varphi$ is the internal friction angle, and $C$ is the cohesion.

By substituting Eq. (2) into Eq. (1), we obtain

3 $$ \frac{{d\sigma_{r}^{p} }}{{C \cdot \cot \varphi + \sigma_{r}^{p} }} = \frac{2\sin \varphi }{{1 - \sin \varphi }} \cdot \frac{dr}{r} $$

By integrating Eq. (3), Eq. (4) is obtained.

4 $$ \sigma_{r}^{p} + C \cdot \cot \varphi = Ar^{{\frac{2\sin \varphi }{{1 - \sin \varphi }}}} $$

On the wall of the roadway, $r = r_{0}$ and $\sigma_{r}^{p} = p_{i} (t)$. $r_{0}$ is the radius of the roadway and $p_{i} (t)$ is the support resistance changing with time. According to this boundary condition, we can obtain

5 $$ A = (p_{i} (t) + C \cdot \cot \varphi )r_{0}^{{\frac{ - 2\sin \varphi }{{1 - \sin \varphi }}}} $$

Substituting Eq. (5) back into Eqs. (4), (6) can be obtained from Eq. (2).

6 $$ \left\{ \begin{gathered} \sigma_{r}^{p} = (p_{i} (t) + C \cdot \cot \varphi )\left( {\frac{r}{{r_{0} }}} \right)^{{\frac{2\sin \varphi }{{1 - \sin \varphi }}}} - C \cdot \cot \varphi \hfill \\ \sigma_{\theta }^{p} = (p_{i} (t) + C \cdot \cot \varphi ) \cdot \frac{1 + \sin \varphi }{{1 - \sin \varphi }} \cdot \left( {\frac{r}{{r_{0} }}} \right)^{{\frac{2\sin \varphi }{{1 - \sin \varphi }}}} - C \cdot \cot \varphi \hfill \\ \end{gathered} \right. $$

(2) Stress distribution in the viscoelastic zone

The constitutive equation of viscoelastic zone adopts the Poynting–Thomson standard linear solid model. The physical equation of the viscoelastic zone then can be defined by

7 $$ S_{ij} + \tau \mathop {S_{ij} }\limits^{ \bullet } = 2G_{\infty } e_{ij} + 2\tau G_{0} \mathop e\limits^{ \bullet }_{ij} $$

where $S_{ij}$ is the partial stress tensor; $\mathop {S_{ij} }\limits^{ \bullet }$ is the time derivative of the stress partial tensor; $\tau$ is the relaxation time; $G_{\infty }$ and $G_{0}$ are the long-term and instantaneous shear moduli respectively; $e_{ij}$ is the partial strain tensor; and $\mathop e\limits^{ \bullet }_{ij}$ is the time derivative of the strain partial tensor.

8 $$ \left\{ \begin{gathered} S_{ij} = \sigma_{ij} - \sigma_{m} \delta_{ij} \hfill \\ e_{ij} = \varepsilon_{ij} - \varepsilon_{m} \delta_{ij} \hfill \\ \sigma_{m} = \frac{1}{3}\sigma_{kk} = \frac{1}{3}(\sigma_{r} + \sigma_{\theta } + \sigma_{z} ) \hfill \\ \varepsilon_{m} = \frac{1}{3}\varepsilon_{kk} = \frac{1}{3}(\varepsilon_{r} + \varepsilon_{\theta } ) \hfill \\ \end{gathered} \right. $$

where $\sigma_{ij}$ is the stress tensor, $\sigma_{m}$ is the average stress, $\sigma_{kk}$ is the body stress, $\varepsilon_{ij}$ is the strain tensor, $\varepsilon_{m}$ is the average strain, and $\delta_{ij}$ is the Labradoroperator.

Regardless of the size of the plastic zone and whether or not there is support resistance, the stress at the interface of the viscoelastic zone and the plastic zone is constant. Let the calculated point $r = aR_{0} (t)$, $a$ be the scale factor, and $R_{0} (t)$ the radius of the plastic zone changing with time. For the point changing with the radius of the plastic zone, the stress state will not change with time. Therefore, the term for stress rate in Eq. (7) is 0, and the average strain and average strain rate are considered to be 0. Equation 7 then can be reduced to

9 $$ \left\{ \begin{gathered} \sigma_{r} - P = 2G_{\infty } \varepsilon_{r} + 2\eta_{ret} G_{\infty } \mathop {\varepsilon_{r} }\limits^{ \bullet } \hfill \\ \sigma_{\theta } - P = 2G_{\infty } \varepsilon_{\theta } + 2\eta_{ret} G_{\infty } \mathop {\varepsilon_{\theta } }\limits^{ \bullet } \hfill \\ \end{gathered} \right. $$

where $\eta_{ret}$ is the delay time, $\eta_{ret} = \tau G_{0} /G_{\infty }$, and $P$ is the original rock stress.

In addition, in the case of axisymmetry, the volumetric deformation of the viscoelastic region is 0; according to the foregoing assumption, that is

10 $$ \left\{ \begin{gathered} \varepsilon_{m} = \frac{1}{3}\varepsilon_{kk} = \frac{1}{3}(\varepsilon_{r} + \varepsilon_{\theta } ) \hfill \\ \varepsilon_{r} = \frac{du}{{dr}} \hfill \\ \varepsilon_{\theta } = \frac{u}{r} \hfill \\ \end{gathered} \right. $$

Then, according to Eq. (10), we can obtain

11 $$ u = \frac{A(t)}{r} $$

where $u$ is the displacement, and $A(t)$ is a function of time.

Since the plastic zone changes with time, let $r = R_{0} (t)$ in Eq. (11). We then obtain

12 $$ \left\{ \begin{gathered} \varepsilon_{r} = \frac{du}{{dr}} = - \frac{A(t)}{{R_{0}^{2} (t)}} \hfill \\ \varepsilon_{\theta } = \frac{u}{r} = \frac{A(t)}{{R_{0}^{2} (t)}} \hfill \\ \frac{{d\varepsilon_{r} }}{dt} = - \frac{1}{{R^{2}_{0} (t)}} \cdot \frac{dA(t)}{{dt}} + \frac{2A(t)}{{R^{3}_{0} (t)}}\frac{{dR_{0} (t)}}{dt} \hfill \\ \frac{{d\varepsilon_{\theta } }}{dt} = \frac{1}{{R^{2}_{0} (t)}} \cdot \frac{dA(t)}{{dt}} - \frac{2A(t)}{{R^{3}_{0} (t)}}\frac{{dR_{0} (t)}}{dt} \hfill \\ \end{gathered} \right. $$

By substituting the first and third formulas of Eq. (12) into the first formula of Eq. (9), and considering that $\sigma_{r} = \sigma_{{R_{0} }} = P(1 - \sin \varphi ) - C\cos \varphi$, Eq. (13) can be obtained.

13 $$ \left\{ \begin{gathered} \frac{dA(t)}{{dt}} + \left( {\frac{1}{{\eta_{ret} }} - \frac{2}{{R_{0} (t)}} \cdot \frac{{dR_{0} (t)}}{dt}} \right) \cdot A(t) = \frac{{MR_{0} (t)}}{{4G_{\infty } \eta_{ret} }} \hfill \\ M = 2(P - \sigma_{{R_{0} }} ) = 2(P\sin \varphi + C\cos \varphi ) \hfill \\ \end{gathered} \right. $$

When t = 0, $A(0) = \frac{{MR_{0}^{2} (0)}}{{4G_{0} }}$ and substituting this into Eq. (13), we can obtain the general solution of Eq. (13).

14 $$ A(t) = \frac{{MR_{0}^{2} (t)}}{4}\left[ {\frac{1}{{G_{\infty } }} - \left( {\frac{1}{{G_{\infty } }} - \frac{1}{{G_{0} }}} \right)\exp ( - \frac{t}{{\eta_{ret} }})} \right] $$

Substituting Eq. (14) into Eqs. (9), (11), and (12), respectively, the displacement and stress distribution of the viscoelastic region can be obtained.

15 $$ \left\{ \begin{gathered} u_{r}^{c} = \frac{{MR_{0}^{2} (t)}}{4r}\left[ {\frac{1}{{G_{\infty } }} - \left( {\frac{1}{{G_{\infty } }} - \frac{1}{{G_{0} }}} \right)\exp ( - \frac{t}{{\eta_{ret} }})} \right] \hfill \\ \sigma_{r}^{c} = P - \frac{{MR_{0}^{2} (t)}}{{2r^{2} }} \hfill \\ \sigma_{\theta }^{c} = P + \frac{{MR_{0}^{2} (t)}}{{2r^{2} }} \hfill \\ \end{gathered} \right. $$

where $u_{r}^{c}$ is the displacement of the viscoelastic zone; $\sigma_{r}^{c}$ and $\sigma_{\theta }^{c}$ are the radial stress and tangential stress of the viscoelastic zone, respectively, and the letter $c$ represents the abbreviation for creep.

Since Eq. (15) calculates the displacement and stress distribution of the viscoelastic region, r > $R_{0} (t)$ in Eq. (15).

(3) Plastic zone radius under creep conditions

According to the assumption of Eq. (10), the volume strain of the plastic zone is also 0, and the displacement of plastic zone can be calculated by reference to Eq. (11).

16 $$ u_{r}^{p} = \frac{A^{\prime}(t)}{r} $$

When $r = R_{0} (t)$, we can obtain

17 $$ u_{{R_{0} (t)}}^{p} = u_{{R_{0} (t)}}^{c} = \frac{A^{\prime}(t)}{{R_{0} (t)}} = \frac{{MR_{0}^{2} (t)}}{{4R_{0} (t)}}\left[ {\frac{1}{{G_{\infty } }} - \left( {\frac{1}{{G_{\infty } }} - \frac{1}{{G_{0} }}} \right)\exp ( - \frac{t}{{\eta_{ret} }})} \right] $$

Therefore,

18 $$ A^{\prime}(t) = \frac{{MR_{0}^{2} (t)}}{4}\left[ {\frac{1}{{G_{\infty } }} - \left( {\frac{1}{{G_{\infty } }} - \frac{1}{{G_{0} }}} \right)\exp ( - \frac{t}{{\eta_{ret} }})} \right] $$

Substituting Eq. (18) into Eq. (16), we can obtain

19 $$ u_{r}^{p} = \frac{{MR_{0}^{2} (t)}}{4r}\left[ {\frac{1}{{G_{\infty } }} - \left( {\frac{1}{{G_{\infty } }} - \frac{1}{{G_{0} }}} \right)\exp ( - \frac{t}{{\eta_{ret} }})} \right] $$

Equation 19 is similar to Eq. (15) but is different here in that r < $R_{0} (t)$.

According to Eq. (19), the displacement of the roadway wall can be calculated. At this time, $r = r_{0}$. Thus, we obtain

20 $$ u_{{r_{0} }}^{p} (t) = \frac{{MR_{0}^{2} (t)}}{{4r_{0} }}\left[ {\frac{1}{{G_{\infty } }} - \left( {\frac{1}{{G_{\infty } }} - \frac{1}{{G_{0} }}} \right)\exp ( - \frac{t}{{\eta_{ret} }})} \right] $$

From Eq. (20), the range of plastic zones varying with time can be solved.

21 $$ R_{0} (t) = \left[ {\frac{{4r_{0} u_{{r_{0} }}^{p} (t)}}{{M\left( {\frac{1}{{G_{\infty } }} - \left( {\frac{1}{{G_{\infty } }} - \frac{1}{{G_{0} }}} \right)\exp ( - \frac{t}{{\eta_{ret} }})} \right)}}} \right]^{\frac{1}{2}} $$

When calculating the radius of the plastic zone as a function of time according to Eq. (21), it is necessary to measure the displacement value of the wall of the roadway with time.

Equation (21) represents the case of no support. If the support resistance is considered, the support resistance is expressed by Eq. (22).

22 $$ p_{i} (t) = (P + C\cot \varphi )(1 - \sin \varphi )\left( {\frac{{r_{0} }}{{R_{0} (t)}}} \right)^{{\frac{2\sin \varphi }{{1 - \sin \varphi }}}} - C\cot \varphi $$

where $p_{i} (t)$ is the support resistance.

The radius of the plastic zone as a function of time can be obtained from Eq. (22) and can be expressed by Eq. (23).

23 $$ R_{0} (t) = r_{0} \left( {\frac{(P + C\cot \varphi )(1 - \sin \varphi )}{{p_{i} (t) + C\cot \varphi }}} \right)^{{\frac{1 - \sin \varphi }{{2\sin \varphi }}}} $$

To calculate the radius of the plastic zone in Eq. (23), the relationship between support resistance and time needs to be measured. The support resistance increases with time and the increase in support resistance can prevent the deformation of the plastic zone. This paper considers the most unfavorable situation, that is, the existence of unsupported resistance. If the support is not considered, Eq. (23) can be replaced by the following equation when there is no measured displacement of the roadway wall.

24 $$ R_{0} (t) = r_{0} \left( {\frac{{\sqrt 6 \left( {P - \frac{{\delta \cdot K_{p} }}{\sqrt 6 }\left( {1 - \left( {1 - \frac{1}{\delta }} \right) \cdot \exp ( - k \cdot t)} \right)} \right)}}{{2 \cdot \delta \cdot K_{p} \left( {1 - \left( {1 - \frac{1}{\delta }} \right) \cdot \exp ( - k \cdot t)} \right)}}} \right) $$

where $\delta$ is the long-term strength reduction coefficient of rock, $K_{p} = 1.5P$ and $k$ is the roadway relaxation time.

Equation (24) is a time-dependent model of the plastic zone radius under creep conditions.

## Model application

### Application background

**Figure 2:**
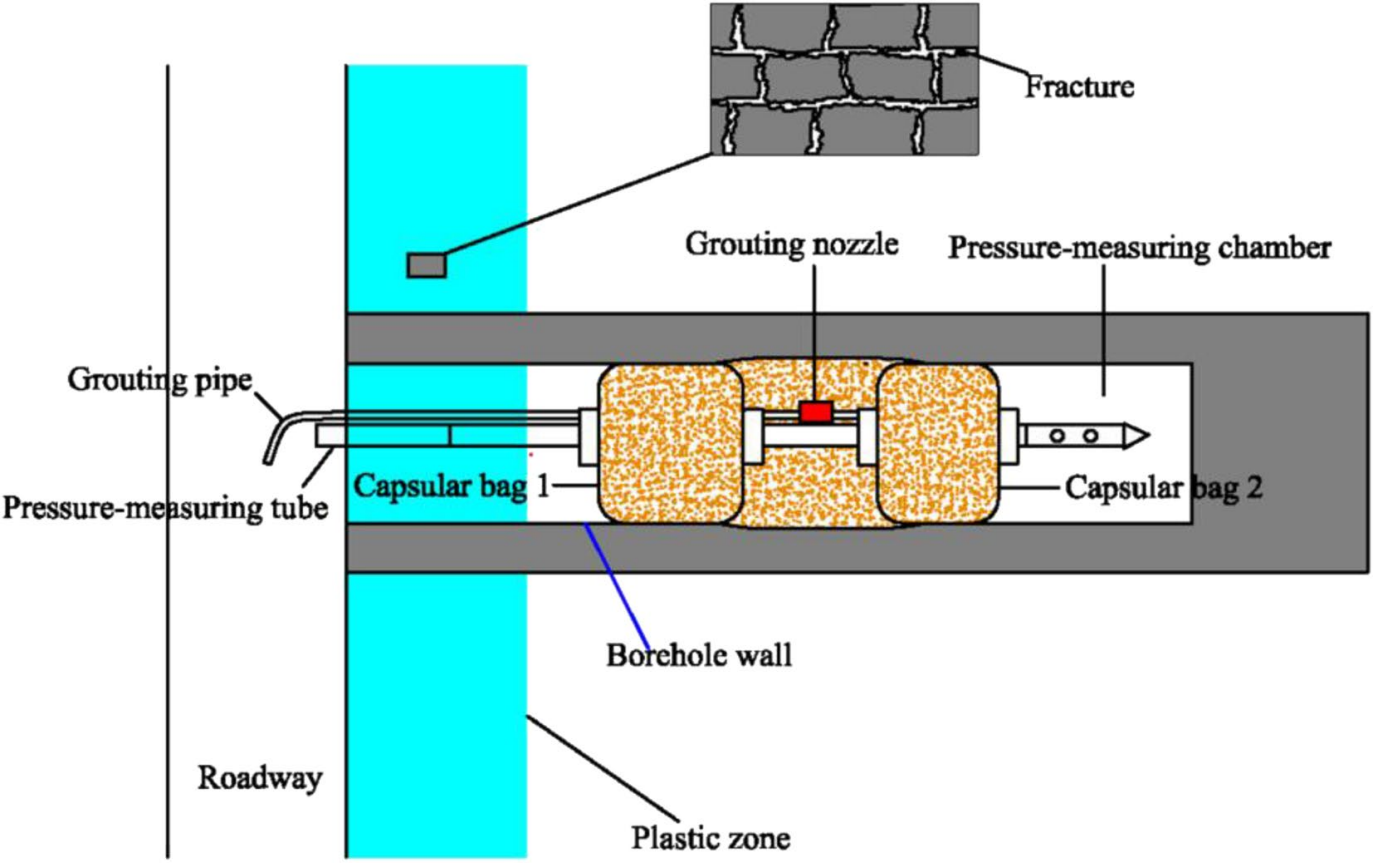
Schematic diagram of the bag-type grouting sealing method^21,22^.

The gas pressure of a coal seam is one of the important indexes for the prediction of the risk of coal and gas outbursts in coal mines. The bag-type grouting sealing method (Fig. 2) is a commonly used method to measure the gas pressure of coal seams in China. The test principle is as follows. When the capsular bags 1 and 2 are delivered to a predetermined position, the grouting pipe is used to grout the bag. After the capsular bags 1 and 2 expand, the bags and the borehole wall are in close contact. Then, with the grouting pressure exceeding the opening pressure of the grouting nozzle, the space between the two capsular bags is injected with grout to form a pressure chamber.

The key to accurately determining the gas pressure of the coal seam by the capsular bag grouting method is finding the range of the plastic zone of the roadway. If capsular bag 1 and capsular bag 2 are located in the plastic zone of roadway, the gas in the pressure-measuring chamber may leak out along the borehole wall and through cracks in the plastic zone during the pressure measurement, and the measured gas pressure will thus be smaller than the actual value or no indication on the pressure gauge. Conversely, when the positions of capsular bag 1 and capsular bag 2 are outside the plastic zone of roadway, the pressure-measuring chamber is ensured to be airtight and the correct gas pressure value will be obtained. Therefore, the accuracy of the calculated plastic zone of roadway can be approximately reflected by the measured value of the gas pressure.

### Plastic zone range of a roadway at the experimental coal mine

The 11111 workface of the No. 13 Coal Mine of Pingdingshan Tianan Coal Mining Corporation Limited in the Henna province of China was selected as the research background. This specific coal mine is classified as a coal and gas outburst coal mine. The workface has an average thickness of 5.85 m and a coal seam inclination of 10°–19°. Considering the danger of coal and gas outburst, a floor rock roadway was used to construct the boreholes to drain the gas from the 11111 workface. The height and width of the roadway are 3 m and 4 m, respectively. The lithology of the floor rock roadway is marlstone and the roadway is 12–19 m away from the coal floor.

**Figure 3:**
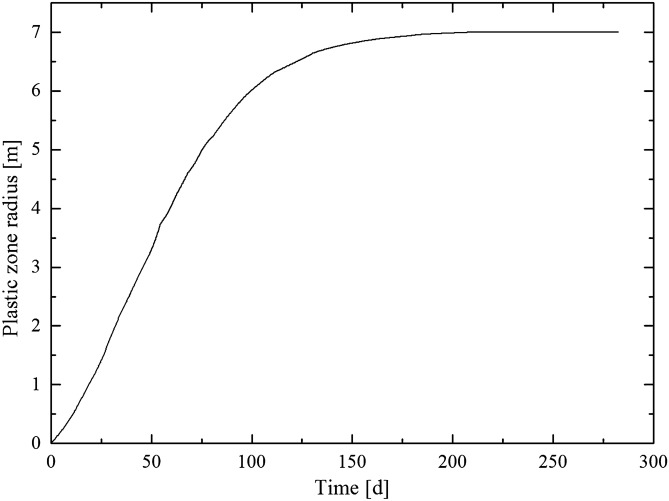
Relationship between the plastic zone radius of the roadway and time.

The buried depth of the floor rock roadway is approximately 600 m. The stress of the floor rock roadway is 15 MPa. The long-term strength reduction coefficient of marlstone is 0.37^23^. According to laboratory measurement, the relaxation time of marlstone is 1/24 d. By substituting the above parameters into Eq. (24), the relationship between the plastic zone radius of the floor rock roadway and time can be obtained, as shown in Fig. 3. Before the 100th day of the formation of the roadway, the plastic zone develops rapidly. During the 100th–150th day of roadway formation, the plastic zone development begins to slow down. After the 200th day of roadway formation, the plastic zone is basically stable and no longer expanding, and the radius of the plastic zone is approximately 7 m.

Therefore, during the sealing of the gas-pressure-measuring borehole for the 11111 workface, the sealing position of the starting capsular bag (capsular bag 1) should be at least 7 m away from the borehole orifice.

### Gas pressure measuring at the experimental coal mine

**Figure 4:**
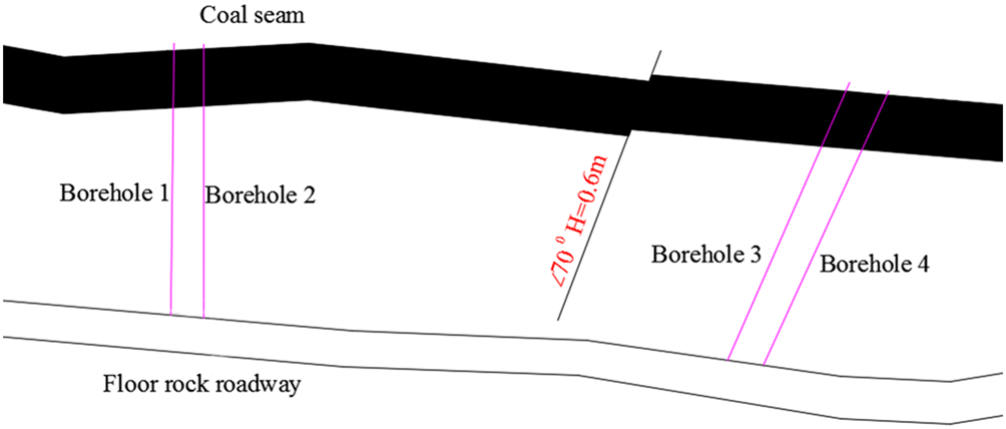
Specific position of the gas pressure measuring borehole at the 11111 workface.

The specific position of the gas pressure measuring borehole at the 11111 workface is shown in Fig. 4. There are 4 boreholes, and the boreholes are named borehole 1–borehole 4.

**Figure 5:**
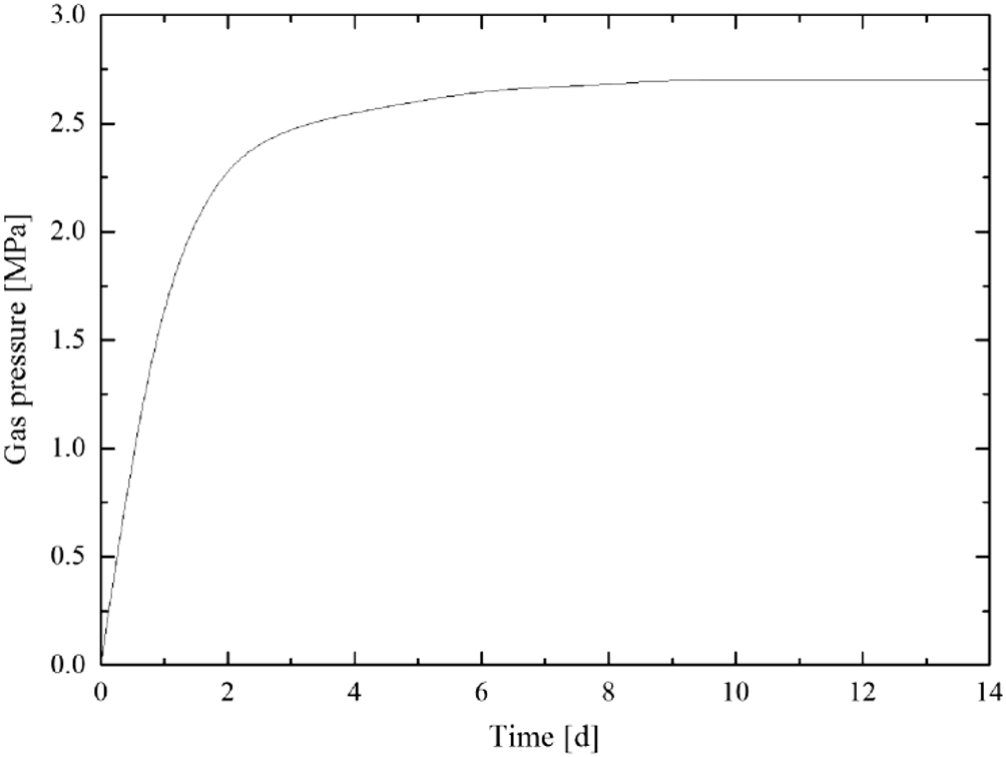
Gas pressure recovery curve of borehole 1.

**Figure 6:**
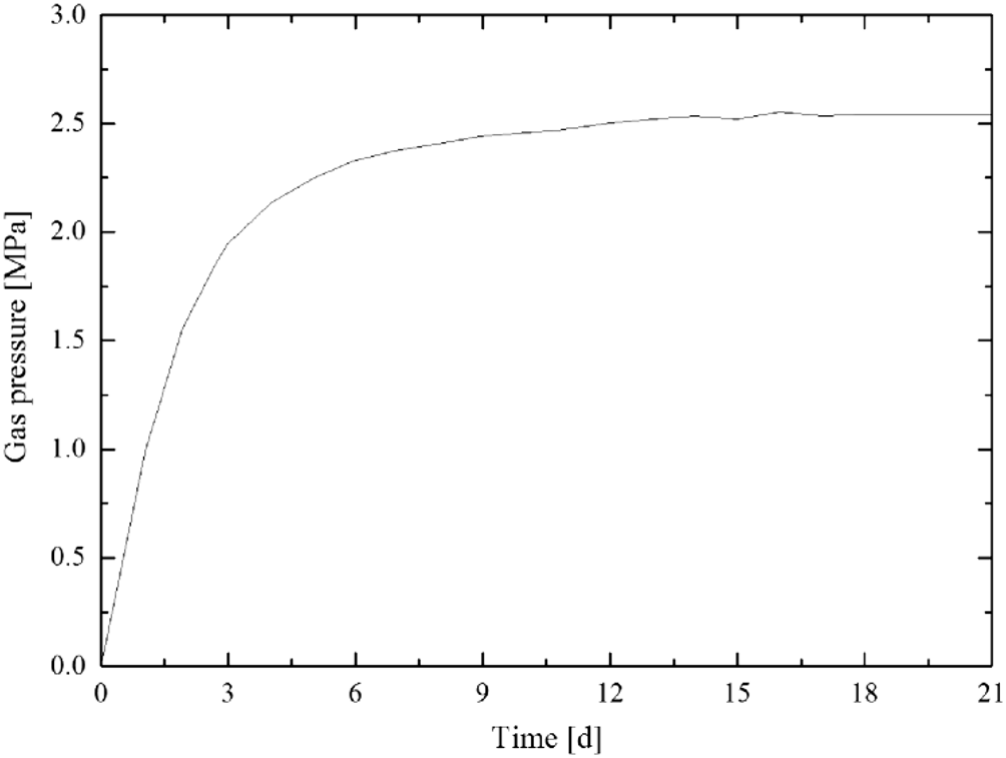
Gas pressure recovery curve of borehole 2.

**Figure 7:**
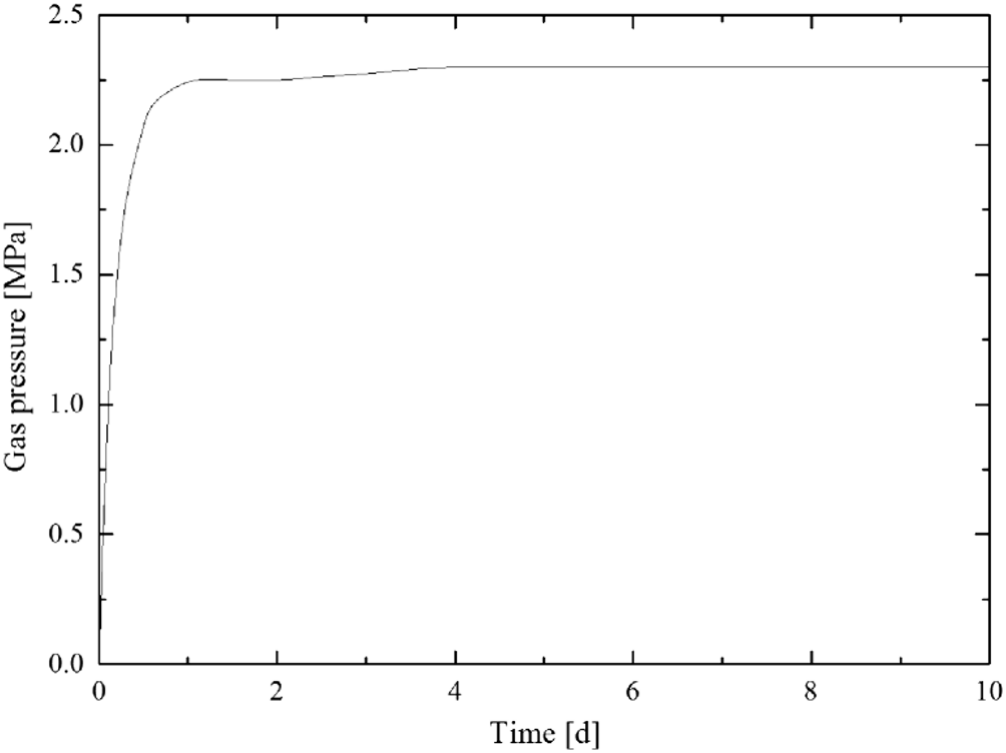
Gas pressure recovery curve of borehole 3.

**Figure 8:**
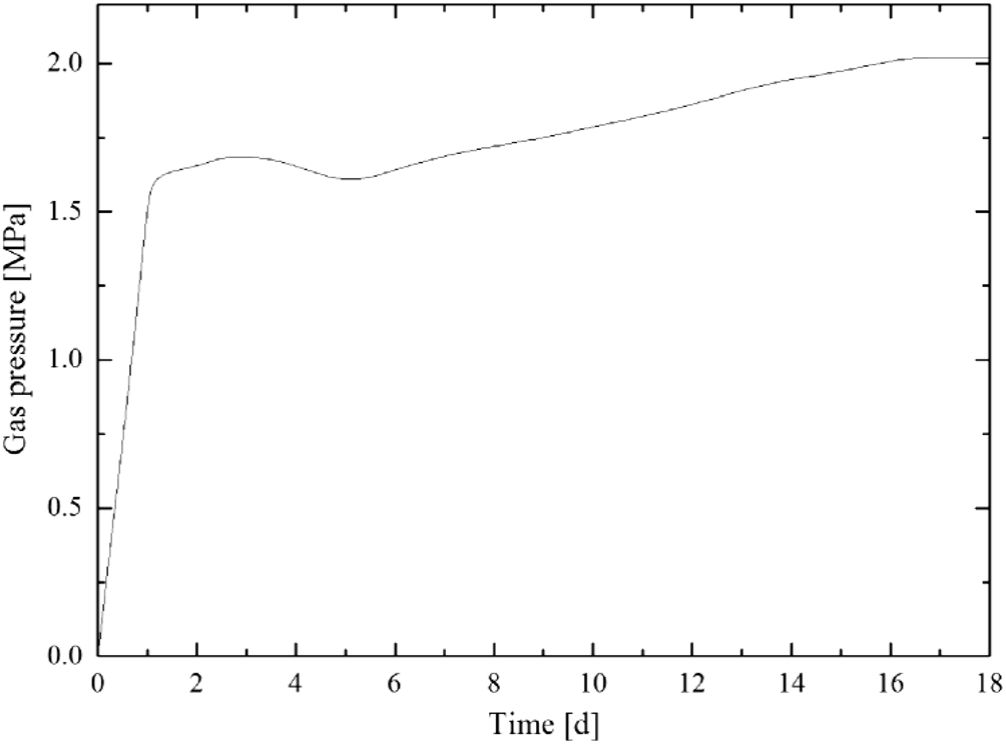
Gas pressure recovery curve of borehole 4.

According to the calculation result of the plastic zone radius of the floor rock roadway, considering the certain margin coefficient, the starting capsular bag (capsular bag 1) is designed to be 8 m away from the borehole orifice when the pressure borehole is sealed. The gas pressure recovery curve measured by the pressure measurement process is shown in Figs. 5, 6, 7, 8. The final gas pressures of boreholes 1–4 are 2.71 MPa, 2.54 MPa, 2.30 MPa and 2.02 MPa, respectively.

In the process of coal seam gas pressure measurement, the direct gas content determination method was used to measure the gas content of the coal seam and the measurement results are shown in Table 1.

**Table 1:** Inverse and measured values of gas content.

Borehole number	Gas pressure (MPa)	Inverse values of gas content (m^3^/t)	Measured values of gas content (m^3^/t)
Borehole 1	2.71	14.98	14.12
Borehole 2	2.54	14.63	13.92
Borehole 3	2.30	14.09	13.68
Borehole 4	2.02	13.36	12.49

In addition, there is the following relationship between gas pressure and gas content^24^.

25 $$ W = \frac{abP}{{1 + bP}} \times \frac{100 - Ad - Mad}{{100}} \times \frac{1}{1 + 0.31Mad} + \frac{10\pi P}{\gamma } $$

where $W$ is the gas content, m^3^/t; $a$ is the adsorption constant, 33.333 m^3^/t; $b$ is also the adsorption constant, 0.767 MPa^−1^; $P$ is the absolute gas pressure of the coal seam, MPa; $Ad$ is the ash content of the coal, 9.00%; $Mad$ is the moisture content of the coal, 1.56%; $\pi$ is the porosity of the coal, 0.0637 m^3^/m^3^; and $\gamma$ is the density of the coal, 1.42 t/m^3^.

Combined with the gas pressure, the gas content can be inversely calculated according to Eq. (25). The inverse calculation results are shown in Table 1.

It can be seen from Table 1 that the gas content calculated by the coal seam gas pressure is consistent with the measured gas content with a maximum phase difference of only 0.87 m^3^/t. This indicates that the measured value of the gas pressure is accurate and can correctly reflect the gas occurrence characteristics of the 11111 workface. Therefore, the validity of the plastic zone calculation model is also verified.

## Conclusions

In this paper, a plastic zone model considering the creep effect is proposed. The model has good applicability and can be used to calculate the plastic zone under different in situ stress conditions and different roadway sizes.

According to the calculation model for the plastic zone, the radius of the plastic zone of a roadway in the No. 13 Coal Mine of Pingdingshan Tianan Coal Mining Corporation Limited in China is 7 m. Based on the radius of the plastic zone of the roadway, the starting capsular bag is designed to be 8 m away from the borehole orifice when the gas pressure measurement borehole is sealed in the roadway. The final measured gas pressure is 2.02–2.70 MPa. In the process of coal seam gas pressure measurement, the direct gas content determination method was used to measure the gas content of the coal seam. In addition, combined with gas pressure, the gas content can be inversely calculated. The gas content thus calculated from the coal seam gas pressure is consistent with the measured gas content, indicating that the gas pressure measurement value is accurate. The gas pressure measurement results verify the validity of the plastic zone calculation model.

## Data Availability

All data used to support the findings of this study are available from the corresponding author upon request.
